# Smart Antidiabetic Nanomedicine: A Revolutionized Therapeutic Approach for Treatment of Diabetes Mellitus

**DOI:** 10.3390/bioengineering12121309

**Published:** 2025-11-28

**Authors:** Alireza Mohammad Karim

**Affiliations:** Division of Cardiology, Department of Pediatrics, Children’s Hospital of Philadelphia, Philadelphia, PA 19104, USA; am2633@cantab.ac.uk

**Keywords:** nanomedicine, self-assembly, diabetes, fibril, peptide

## Abstract

Diabetes mellitus affects over 530 million adults globally, with current therapies limited by frequent dosing, <20% oral bioavailability, and poor long-term glycemic control. Nanomedicine, particularly self-assembled peptide nanostructures (nanofibrils), offers sustained, glucose-responsive drug release and extended peptide bioactivity for several days per dose. This review critically evaluates recent advances in smart antidiabetic nanomedicine, focusing on quantitative improvements in pharmacokinetics, controlled release, and patient compliance compared with conventional treatments. It also outlines remaining challenges in large-scale synthesis, safety validation, and regulatory translation. Collectively, these insights highlight the potential of reversible peptide nanofibrils as long-acting, cost-effective therapeutics for improved diabetes management.

## 1. Introduction

### 1.1. Diabetes Mellitus and Its Complications

**Figure 1:**
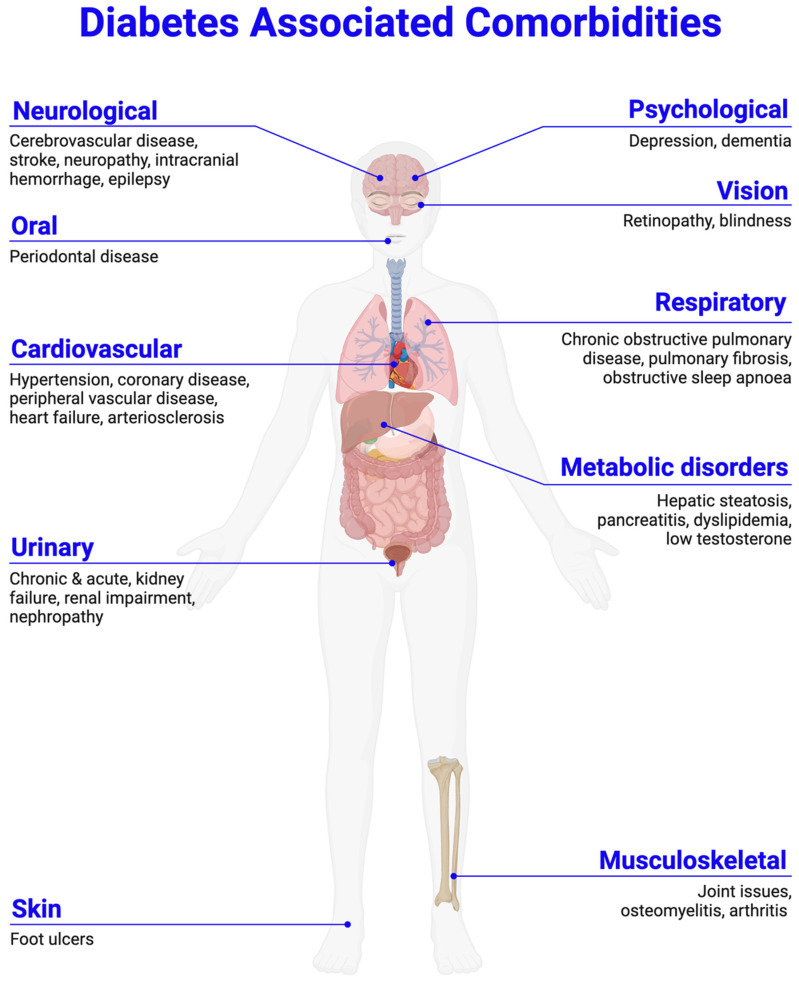
Diabetes mellitus frequently occurs concurrently with other health complications, including neurological, psychological, oral, vision, cardiovascular, respiratory, metabolic disorders, renal, and rheumatological disorders. Created in BioRender. Mohammad Karim, A. (2025) https://BioRender.com/vyps8x4. Accessed on 13 November 2025.

Diabetes mellitus comprises a group of metabolic disorders characterized by hyperglycemia resulting from impaired insulin secretion or activity [1,2,3,4]. The International Diabetes Federation projects that by 2045, approximately 693 million individuals will be affected worldwide [5]. Chronic hyperglycemia leads to dysfunction and complications across multiple organ systems—including visual, neurological, rheumatological, respiratory, digestive, renal, and cardiovascular (Figure 1) [1,2,3,4].

Diabetes mellitus is broadly classified into type 1 (T1DM) and type 2 (T2DM) [6]. T1DM, accounting for 5–10% of cases, arises from autoimmune destruction of pancreatic β-cells, resulting in absolute insulin deficiency [1,2,3,4,7]. In contrast, T2DM, representing 90–95% of cases, involves insulin resistance and inadequate compensatory insulin secretion [1,2,3,4,8].

Diabetic patients are often overweight, a major contributor to insulin resistance. Long-term diabetes leads to micro- and macrovascular complications, including diabetic retinopathy, nephropathy, neuropathy, cardiomyopathy, foot ulcers, cerebrovascular disease, bladder dysfunction, and erectile dysfunction [9,10,11,12,13,14,15,16,17,18].

### 1.2. Conventional Therapies for Diabetes Mellitus and Their Drawbacks

Diet and exercise are primary measures to control hyperglycemia and prevent diabetes onset [19,20]. When these fail, patients rely on oral antidiabetic drugs or insulin injections. Major therapeutic classes include biguanides (metformin), sulfonylureas (acetohexamide, metahexamide, glyclopyramide), DPP-IV inhibitors (vildagliptin, linagliptin), incretin analogs (liraglutide, semaglutide, exenatide, dulaglutide), thiazolidinediones (pioglitazone), and SGLT2 inhibitors (dapagliflozin) [21,22,23,24,25,26,27,28]. These agents regulate blood glucose by activating the GLP-1 receptor (GLP-1R) to enhance insulin secretion and reduce body weight [29,30]. However, such drugs require frequent or lifelong injections, which can induce hypoglycemia, seizures, or even death in severe cases [21,31].

**Figure 2:**
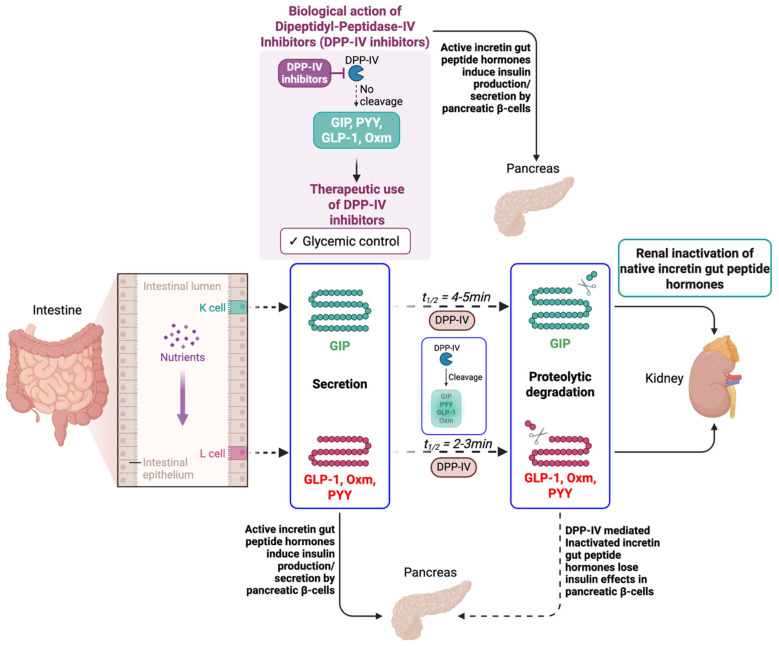
The schematic diagram depicts the mechanisms of metabolic and proteolytic degradation of incretin gut peptide hormones (GIP, PYY, GLP-1, and Oxm). The role of DPP-IV is to bypass the proteolytic degradation effect of DPP-IV on the gut peptide hormones to induce insulin production and secretion by pancreatic β-cells. Created in BioRender. Mohammad Karim, A. (2025) https://BioRender.com/rgqaoj0. Accessed on 13 November 2025.

After Roux-en-Y gastric bypass (RYGB)**,** patients with T2DM and obesity exhibit increased secretion of gut incretin peptides—PYY, GIP, GLP-1, and oxyntomodulin (Oxm)—that reduce food intake, delay gastric emptying, and enhance nutrient uptake [32,33,34]. These hormones coordinate insulin and glucagon secretion while suppressing appetite [32,35]. Yet, their antidiabetic efficacy is limited by poor biochemical stability and rapid degradation by DPP-IV, leading to low oral bioavailability (Figure 2). To overcome this, multiple DPP-IV inhibitors have been developed and studied to prolong incretin activity [26,36].

Oral antidiabetic drugs face major limitations, including gastric irritation, diarrhea, low solubility, short half-life, and frequent dosing due to poor bioavailability [19]. They also fail to resist biochemical and proteolytic degradation upon exposure to gastrointestinal barriers, compromising stability and bioactivity under physiological and pathological conditions [37,38].

Subcutaneous insulin therapy requires continuous glucose monitoring and multiple daily injections, often causing pain, poor compliance, and risks of hyperglycemia or hypoglycemia [31,39]. Even with “closed-loop” insulin pumps, delayed or inconsistent glucose regulation remains a serious clinical challenge [40,41]. Therefore, there is an urgent need for precise and responsive alternatives. Nanomedicine offers significant promise for improving the accuracy, stability, and timeliness of diabetes management [42,43].

This review aims to consolidate recent progress in antidiabetic nanomedicine, with emphasis on self-assembled peptide-based nanostructures (nanofibrils) as reversible and long-acting therapeutic systems. Although previous reviews have broadly discussed nanotechnology applications in diabetes [19,20,21,44], few have critically examined the mechanistic and translational aspects of peptide-based nanomedicine. This review, therefore, provides an integrated perspective on their structural basis, therapeutic advantages, and challenges toward clinical application.

## 2. Methodology and Search Strategy

This narrative-critical review was conducted through a structured literature search covering 2010–May 2025 across PubMed, Scopus, and Web of Science databases. Search terms included combinations of (“nanomedicine” OR “nanoparticles” OR “self-assembled peptides” OR “nanofibrils”) AND (“diabetes mellitus” OR “type 2 diabetes” OR “insulin delivery” OR “GLP-1” OR “oxyntomodulin”).

Inclusion criteria were peer-reviewed English-language articles focusing on (i) nanotechnology applied to diabetes therapy or diagnostics, (ii) pre-clinical or clinical evaluation of nanocarriers, and (iii) mechanistic or translational relevance. Exclusion criteria included papers lacking original data (e.g., editorials) or those unrelated to metabolic disorders. Reference lists of key papers were cross-checked to identify additional studies. Data synthesis emphasized comparative efficacy, reproducibility, safety, and regulatory readiness.

## 3. Nanomedicine: Promising Approach for Treatment of Diabetes Mellitus

Nanomedicine, the medical application of nanomaterials, enables controlled and targeted drug delivery to enhance therapeutic efficacy through novel nanocarrier systems [20,42,45,46,47,48]. It offers a promising strategy to overcome the limitations of conventional antidiabetic therapies [49]. The integration of nanoscience and biomedical engineering has advanced diabetes management by improving drug delivery platforms and developing bio–nanosensors for real-time glucose monitoring [50,51]. Sensitive non-enzymatic metal oxide glucose nanosensors can continuously detect glucose in sweat or tears, enhancing patient quality of life [52].

In diabetic wound care, nanomedicine promotes healing by regulating hemostasis, infection, inflammation, and tissue regeneration [53,54,55,56,57]. Effective nanotherapeutic systems include silk-based nanomaterials, nanoparticles, nanofibers, nanohydrogels, and scaffolds [55,57,58].

For diabetic cardiovascular complications such as myocardial infarction, stroke, and heart failure, nanotheranostics—combining nanotherapeutics with diagnostic imaging—shows promise for targeted treatment and monitoring of autophagy and mitophagy [59,60,61,62,63].

## 4. Current Nanomedicine Approaches to Treat Diabetes Mellitus

The antidiabetic and insulin nanocarriers employed for the management of diabetes mellitus comprise various types of nanoparticles and self-nanoemulsifying antidiabetic drug delivery systems [64,65,66,67,68,69]. Herbal nanomedicine has also emerged to be a promising approach for the prompt delivery of herbal compounds to individuals with diabetes mellitus [64,65].

### 4.1. Nanoparticles

**Figure 3:**
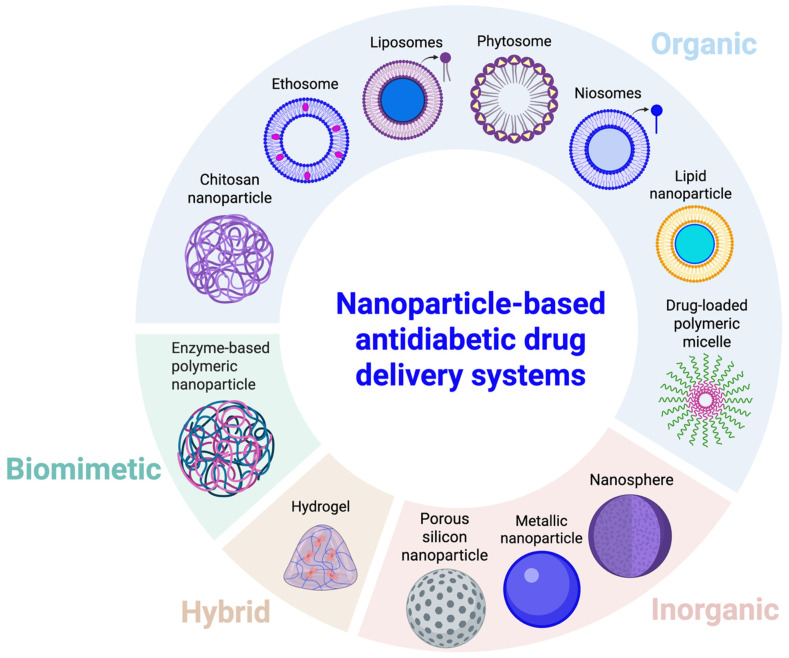
An overview of various antidiabetic nanoparticle-based drug carriers. Organic: composed of polymeric-, lipid-based, and protein-based nanoparticles. Inorganic: comprising metal, metal oxide, and ceramic nanoparticles. Hybrid organic–inorganic: combined features of organic and inorganic nanoparticles. Biomimetic: engineered nanostructures mimicking natural cellular architectures and activities. Created in BioRender. Mohammad Karim, A. (2025) https://BioRender.com/atzvzax. Accessed on 13 November 2025.

In nanomedicine, nanoparticles loaded with antidiabetic drugs can regulate release according to chemo-bio-physiological conditions [21]. Advances in nanoparticle technology have greatly expanded innovative therapies for diabetes mellitus [70,71,72]. Nanocarriers for antidiabetic drugs are generally classified into four main categories (Figure 3): organic (polymeric-, lipid-, and protein-based) [73,74,75,76,77,78], inorganic (metal-based) [79,80,81,82], hybrid (organic–inorganic) [83,84], and biomimetic (cell-derived polymeric) nanoparticles [85,86].

Oral insulin nanotherapeutics provide an effective strategy for diabetes management and improved patient compliance [87,88]. Various nanocarriers have been developed for sustained and targeted insulin delivery in both T1DM and T2DM, including lipid-based (liposomes) [89], polymeric-based (chitosan, alginate, dextran, PLGA) [90,91,92,93,94,95], and inorganic (silica) nanocarriers [96]. Oral nanomedicine enhances insulin absorption, delays its degradation in gastric fluid, and increases bioavailability (up to 14%) for effective glycemic regulation in vivo [88,90].

Recent studies showed that inhalable glycol-chitosan-coated PLGA nanoparticles have strong potential as long-acting inhalable insulin and antidiabetic delivery systems [97,98]. Clinically, intranasal insulin reduces postprandial hyperglycemia [99,100]. *Nasulin™* is a known intranasal formulation [99,100], though adverse effects such as dose-dependent local irritation and hypoglycemia led to FDA rejection [99,100]. *Afrezza™*, a dry-powder, rapid-acting inhaled insulin, is FDA-approved, but causes hypoglycemia, throat irritation, cough, and bronchospasm in patients with asthma or chronic pulmonary disease, with low bioavailability (~24%) [99,100,101,102]. Additionally, GLP-1 peptides have been incorporated into porous silicon and polymeric nanomaterials for oral delivery, protecting the peptide from enzymatic degradation [103,104].

### 4.2. Herbal Nanomedicine

Herbal drugs and phytochemicals have shown potential for diabetes therapy, but their clinical use is limited by poor solubility, bioavailability, and efficacy, leading to suboptimal pharmacokinetics and patient compliance [105]. Nanotechnology offers solutions through targeted delivery systems such as nano-pumps, nanorobots, and nanoscale herbal formulations [106]. Herbal nanomedicines are generally classified as lipid-based or inorganic (metal or metal oxide) nanoparticulate systems, used to deliver plant extracts or phytocompounds to diabetic patients [105,107,108,109,110,111,112,113,114,115,116,117,118,119]. Although these systems improve bioavailability, insufficient preclinical and clinical data still prevent their approval for diabetic therapy [105].

### 4.3. Orderly Self-Assembled Peptide-Based Nanostructures: Smart Antidiabetic Nanomedicine for Treatment of Diabetes Mellitus

Under certain physical conditions, the intrinsic propensity of the peptide backbone and side chain orientations facilitates attractive interactions between the side chains of the homotypic peptides. These attractive interactions stabilize the aggregated conformation of the homotypic peptides as their Gibbs free energy level reaches the minimum value [120]. This process is known as the peptide self-assembly. Peptides can adopt different self-assembled nanostructures, such as a nanofibril, a nanotube, a nano helix, and a nano globular structure [121,122,123,124]. The most common form of the self-assembled conformation to which homotypic peptides aggregate is a nanofibrillar structure. The attractive interactions between the homotypic peptides form infinitely repeating intermolecular β-sheet interactions in a cross-β motif that can elongate by recruiting more homotypic peptides to the ends of the nanofibril [125].

### 4.4. Diabetic Wound Healing

Diabetic wounds, such as foot ulcers, heal slowly and incompletely, leading to a higher infection risk [126]. Nanostructures and smart hydrogels enhance wound repair by promoting peptide self-assembly [127,128]. Puerarin-loaded peptide–copolymer nanocomposite hydrogels accelerated healing in diabetic mice [128], while lipopeptide nanostructures with collagen-stimulating pentapeptides improved wound closure in diabetic rats [129].

Granular hydrogel matrices facilitate mesenchymal stem cell delivery and tissue regeneration through their porous, ECM-like architecture [130]. Engineered nanofibrillar hydrogels formed via hydrogen bonding and hydrophobic interactions improved stem cell retention and anti-inflammatory regulation in diabetic rats, showing strong potential for regenerative therapy [130].

### 4.5. Self-Assembly of Peptides into Nanofibrillar Structures for Prolonged and Controlled Delivery of Drug into Diabetic Patients

**Figure 4:**
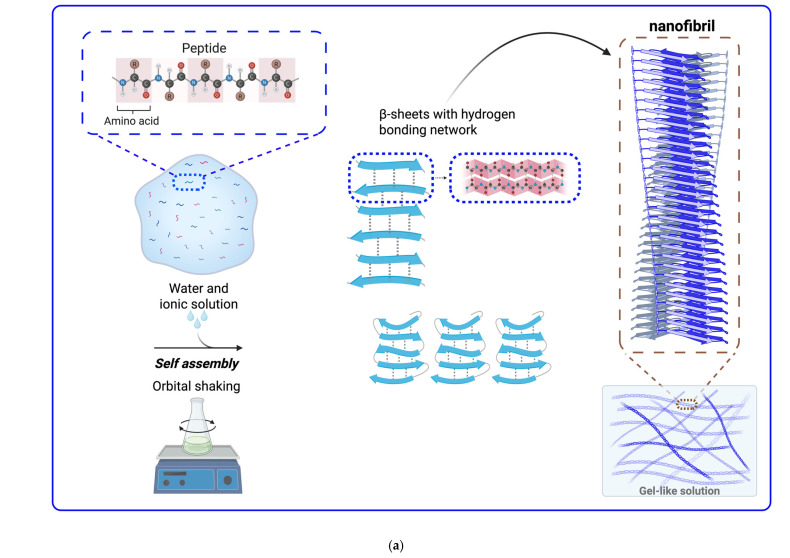

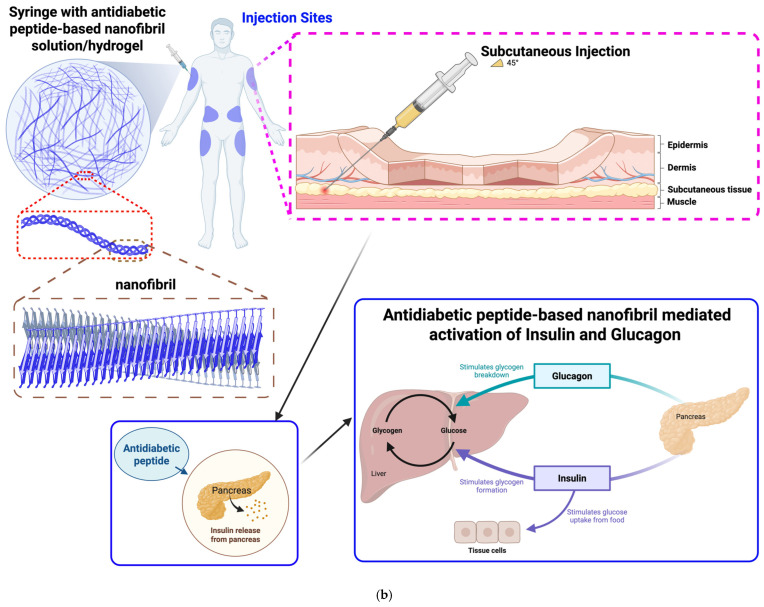
(**a**) Process of self-assembly of peptides (i.e., antidiabetic peptides) into a nanofibrillar conformation. The hydrogen bonding network between the peptides (dotted lines) induces incorporation of the β-sheets, and the intermolecular interactions between the β-sheets, such as ionic, polar, and hydrophobic forces between residues, promote attractions between the β-sheets towards formation of a nanofibril. (**b**) Subcutaneous injection of antidiabetic peptides based on nanofibril hydrogel or solution in diabetic patients induces antidiabetic activities in vivo by regulating pancreatic glucagon and insulin secretions to function biologically in the liver. Created in BioRender. Mohammad Karim, A. (2025) https://BioRender.com/fgvvycq. Mohammad Karim, A. (2025) https://BioRender.com/vse516g. Accessed on 28 November 2025.

The self-assembly of peptides represents a feasible technique to generate biocompatible hydrogels and solutions in the form of suspended nanofibrils to overcome hyperglycemia in diabetic patients. Therefore, the most magnificent way to address diabetes mellitus (T1DM or T2DM) was discovered to be the reversible self-assembly of antidiabetic peptides into nanofibrillar architectures capable of delivering antidiabetic peptides depot through *s.c.* injection in patients and controlled release of the bioactive native free antidiabetic peptides in vivo to drive desired biological responsiveness of the body via biological cellular signaling pathways of glucagon and insulin in the liver (Figure 4). Recent studies explored innovative strategies to form bioactive antidiabetic peptide-based nanofibrils to develop novel antidiabetic drug formulations with regulated pharmacodynamic and pharmacokinetic profiles [131,132,133,134,135].

Recent studies show that human-derived native peptides can enhance drug safety and efficacy, but their clinical application remains limited by challenges in controlling physicochemical and pharmacokinetic stability in vivo [133,136]. Amphiphilic peptides, structured with hydrophobic tails and hydrophilic heads, self-assemble into β-sheet nanofibrillar hydrogels that support human islet bioactivity under hyperglycemic and proinflammatory conditions [137,138]. Nitric oxide (NO)-releasing nanofibrillar hydrogels composed of functionalized peptides promote insulin secretion in β-cells; however, clinical evaluation is required to address risks from uncontrolled NO release [139].

pH- and glucose-responsive β-sheet peptide hydrogels enable glucose-dependent insulin release for up to 12 h without toxicity, though frequent injections limit compliance [140]. Integrating antidiabetic peptides within nanofibrillar hydrogels enhances protection and sustained delivery. For example, RADA16-hempseed hydrolysate hydrogels encapsulating sitagliptin or DPP-IV inhibitory peptides reduced enzymatic degradation and prolonged incretin activity, though cytotoxicity and immunogenicity assessments remain incomplete [36]. Similarly, GLP-1R exendin-2 nanofibrillar hydrogels stimulated insulin production in β-cells [141], and heparin-binding amphiphilic peptide hydrogels delivering VEGF and FGF-2 improved islet engraftment and transient insulin secretion in diabetic models [142,143].

Peptide YY (PYY) nanofibril formulations also showed prolonged appetite suppression compared with liraglutide, yet their long-term safety and efficacy remain undetermined [144]. Overall, antidiabetic peptide-based hydrogels protect β-cells and regulate insulin secretion but require optimization of stability, mechanics, and safety under physiological conditions [145,146].

To address these limitations, oxyntomodulin (Oxm) peptide was developed as a self-assembled nanofibrillar hydrogel with dual GCGR/GLP-1R agonist activity [134]. Oxm nanofibrils provided prolonged, reversible release of active peptides, maintaining bioactivity for several days after a single s.c. injection in rodents A proteolytically resistant analog, Aib2-Oxm, formed slower-growing nanofibrils that released bioactive peptides more efficiently and achieved a five-fold increase in in vivo activity compared with Oxm [135]. Both Oxm and Aib2-Oxm nanofibrils were non-cytotoxic and significantly enhanced plasma exposure, metabolic response, and therapeutic duration for T2DM treatment [134,135].

## 5. Critical Insights and Limitations: From Nanoparticles to Self-Assembled Peptides

Nanomedicine introduces nanoscale drug carriers that improve pharmacokinetics and targeted delivery of insulin and antidiabetic agents [18,19,20,21]. Organic nanoparticles—such as polymeric- (PLGA, chitosan) and lipid-based systems—achieve up to 14% oral insulin bioavailability in vivo, a ten-fold increase over free insulin [22,23]. Inorganic and hybrid nanocarriers add structural stability and sensing capacity but face biodegradation concerns [24]. Comparative analyses reveal that while polymeric nanoparticles enhance absorption, their reproducibility and long-term toxicity remain under-investigated.

A distinct frontier in the field involves self-assembled peptide nanostructures that form β-sheet or helical fibrils acting as smart depots for sustained hormone release [25,26,27,28]. For example, oxyntomodulin-based nanofibrils prolong peptide half-life in rodents from hours to several days [29,30]. However, mechanistic stability under physiological ionic strength and proteolytic stress remains a key translational bottleneck. Critical insights and limitations include the following:

1. Evidence for peptide-based nanomedicine is strong at the in vivo rodent level but absent in human trials.
2. Comparative reproducibility and batch scalability are poorly reported, limiting GMP translation.
3. Future work should couple atomic-level structural characterization (e.g., cryo-EM) with microfluidic organoid testing to bridge bench-to-bedside evaluation.

## 6. Translational and Regulatory Considerations

The successful translation of antidiabetic nanomedicine requires coordinated progress in manufacturing, safety assessment, and regulatory compliance. Scale-up of nanoparticle or nanofibril systems must maintain batch uniformity in particle size, surface charge, and peptide loading. Good Manufacturing Practice (GMP) production typically involves solvent-free processes, defined buffer composition, and endotoxin-free raw materials.

From a regulatory standpoint, agencies such as the Food and Drug Administration (FDA) and European Medicines Agency (EMA) emphasize the following:

- Validated physicochemical characterization;
- Stability testing under physiological pH and temperature;
- Immunogenicity and toxicokinetic profiling in two species before Investigational New Drug (IND) submission.

Table 1 summarizes ongoing clinical investigations in nanomedicine for diabetes, including polymeric oral insulin and lipid-based GLP-1 carriers [93,135]. Despite rapid laboratory progress, regulatory acceptance hinges on demonstrating predictable release kinetics, scalability, and biocompatibility comparable to existing peptide therapeutics. Nanomedicine offers clear advantages over conventional therapies, including targeted and sustained drug release, improved bioavailability, reduced dosing frequency, and better patient compliance. However, challenges such as large-scale production, long-term safety, and regulatory approval remain significant barriers to clinical translation [19,136].

## 7. Clinical Translation, Analytical Evidence, and Commercialization of Nano-Based Antidiabetic Therapeutics

Although nanomedicine has shown strong preclinical potential for diabetes management, the translation of nano-enabled formulations into human trials remains limited. Nevertheless, several nano-based platforms have generated quantitative evidence supporting their therapeutic promise and provide important reference points for future antidiabetic nanomedicine development.

### 7.1. Evidence from Analytical and Preclinical Studies

Multiple studies have provided mechanistic and analytical insights into how nanostructured systems can enhance glycemic control. Ref. [28] reported that oral quantum-dot-based insulin nanocarriers produced a dose-dependent glucose-lowering effect comparable to subcutaneous insulin, while inducing fewer hypoglycemic events and requiring lower overall insulin dosing. These formulations demonstrated prolonged systemic exposure, highlighting the capacity of nano-enabled oral insulin to overcome the low bioavailability (<20%) typically associated with enteral peptide delivery.

Similarly, [153] developed glucose-responsive multivesicular liposomes (MVLs) that released insulin in proportion to ambient glucose levels. In vitro, MVLs exhibited markedly accelerated insulin release at 25 mM glucose relative to normoglycemic conditions, and in vivo, they maintained normoglycemia for extended periods compared with free insulin. Collectively, these studies illustrate how nanoscale engineering improves release kinetics, physiological responsiveness, and pharmacokinetic stability, supporting their relevance as next-generation antidiabetic platforms.

### 7.2. Clinical Studies on Nano-Formulations

Among nano-enabled therapeutics, nano-curcumin remains the most widely tested in humans. A randomized controlled trial involving 70 patients with type 2 diabetes mellitus (T2DM) demonstrated that daily administration of 80 mg nano-curcumin for 3 months produced significant reductions in HbA1c, fasting blood glucose (FBG), triglycerides, and BMI compared with placebo (*p* < 0.05). These findings were corroborated by a meta-analysis of multiple nano-curcumin RCTs, which showed consistent improvements in FBG, fasting insulin, insulin resistance (HOMA-IR), lipid metabolism, and inflammatory biomarkers [154,155,156]. Importantly, these formulations exhibited excellent safety and tolerability profiles, underscoring the feasibility of nano-based bioactive compounds for metabolic disease management.

While peptide-based nanomedicines such as quantum-dot insulin, glucose-responsive liposomes, or peptide-nanofibril depots have advanced through rigorous animal studies, first-in-human pharmacokinetic trials for these systems are still forthcoming. This gap reflects the need for standardized preclinical-to-clinical pathways and robust safety evaluation frameworks tailored to nano-structured peptide systems.

### 7.3. Commercialization Landscape

Only a small number of nano-enabled antidiabetic technologies have achieved clinical or commercial adoption. The most prominent example is Afrezza^®^, an FDA-approved dry-powder inhaled insulin formulated using Technosphere^®^ microparticles composed of fumaryl diketopiperazine nanostructures [157,158]. Clinical trials demonstrated that Afrezza provides rapid pulmonary absorption, non-inferior post-prandial glycemic control, and reduced late hypoglycemia compared with subcutaneous rapid-acting insulin analogs. Despite its pharmacokinetic advantages, widespread adoption has been limited due to device burden, cost, and pulmonary safety monitoring requirements.

In contrast, several nano-curcumin formulations are commercially available as nutraceuticals; however, they are not approved as antidiabetic drugs and lack the regulatory rigor required for pharmaceutical deployment [159]. No nano-enabled insulin, GLP-1 agonist, or peptide-fibril depot has yet reached the regulatory threshold for commercial approval, emphasizing the substantial translational gap that persists despite promising preclinical data [160,161].

### 7.4. Outlook for Translational Advancement

**Figure 5:**
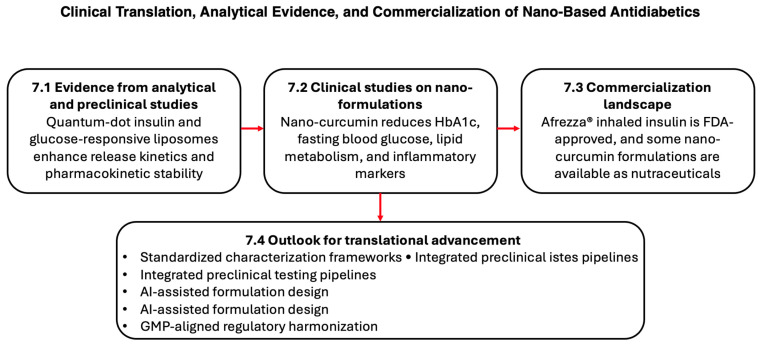
Translational pipeline for smart antidiabetic nanomedicine development. A schematic overview illustrating the stepwise progression of smart antidiabetic nanomedicine—from molecular-level peptide design and self-assembly optimization to physicochemical characterization, preclinical evaluation, scalable manufacturing, regulatory assessment, and clinical translation. The workflow highlights critical checkpoints, including structural elucidation (cryo-EM/helical reconstruction), stability and immunogenicity testing, in vivo pharmacokinetics/pharmacodynamics, GMP-compliant formulation, and development of patient-compliant reversible peptide nanofibril depots. This pipeline emphasizes the multidisciplinary integration required to advance self-assembled peptide nanostructures toward clinically viable antidiabetic therapeutics.

The collective evidence suggests that nano-enabled antidiabetic systems are approaching translational feasibility but require the following (Figure 5):

1. Standardized analytical characterization frameworks to define release kinetics, batch reproducibility, and long-term stability across physiological conditions.
2. Integrated preclinical testing pipelines evaluating immunogenicity, biodistribution, and metabolomic outcomes.
3. AI-assisted formulation design to predict self-assembly behavior, optimize fibril architectures, and reduce manufacturing variability.
4. GMP-aligned regulatory harmonization to bridge academic innovation with scalable industrial production.

By combining these clinical, mechanistic, and regulatory insights, the field is positioned to advance smart antidiabetic nanomedicine from conceptual innovation toward clinically viable and commercially sustainable therapeutic platforms.

## 8. Challenges and Future Perspectives

Nanomedicine represents a cost-effective and patient-compliant platform for managing diabetes mellitus, particularly chronic hyperglycemia and insulin resistance [162,163,164,165]. Synthetic and naturally derived peptide-based self-assembled nanofibrils and non-hydrogels provide drug stability under harsh physiological environments, supporting insulin independence in diabetic patients [162,163,164,165].

Translating these advances into clinical application requires a comprehensive mechanistic understanding of antidiabetic nanomedicine [43]. Integrating pancreatic β-cell 3D cultures and microfluidic organoid models will help elucidate how peptide-based nanofibril hydrogels improve insulin sensitivity and glucose regulation [20]. While current in vivo studies demonstrate good biocompatibility in rodents, potential immunogenic and proinflammatory responses remain unclear [166]. Hence, in silico molecular modeling and standardized immunotoxicity assessments should precede clinical translation [167,168,169].

Nanomedicine offers a transformative avenue for achieving sustained glycemic control through precision-engineered drug carriers that overcome the limitations of current insulin and incretin therapies. Self-assembled peptide-based nanostructures—particularly GLP-1 and Oxm nanofibrils—represent the most promising strategy for developing reversible, long-acting, and patient-compliant antidiabetic depots. Future efforts should prioritize the following:

1. Structural elucidation of peptide nanofibrils at near-atomic resolution using cryo-EM or helical reconstruction to correlate morphology with controlled-release kinetics [170,171].
2. Standardized preclinical testing frameworks evaluating immunogenicity, mechanical robustness, and temperature-dependent stability [146,172,173,174].
3. Integration of AI-assisted formulation design to predict peptide self-assembly behavior, optimize batch reproducibility, and minimize manufacturing variability.
4. Regulatory harmonization bridging academic innovation and industrial production under GMP-compliant conditions to accelerate translation.

Through these multidisciplinary strategies, smart antidiabetic nanomedicine can advance from conceptual innovation to clinically viable, cost-effective therapeutics that improve global diabetes management.

## Figures and Tables

**Table 1:** Comparative features of nanomedicine platforms for diabetes mellitus.

Platform Type	Representative Materials	Key Advantages	Limitations/Challenges	Translational Readiness
Polymeric [93,147,148]	PLGA, chitosan, alginate	Biodegradable; tunable release; oral or injectable use	Batch variability; limited long-term safety data	Pre-clinical → early clinical
Lipid-based [21,43,149]	Liposomes, solid-lipid NPs	High biocompatibility; efficient encapsulation	Instability under storage; low payload	Phase I–II clinical
Inorganic [43,150]	Silica, ZnO, AuNPs	Precise size control; theranostic potential	Poor biodegradation; cytotoxicity	Pre-clinical
Hybrid [151,152]	Polymer–metal oxides	Synergistic mechanical and functional properties	Complex synthesis; cost	Proof-of-concept
Peptide Self-Assembled [21,135]	GLP-1, Oxm, Aib2-Oxm nanofibrils	Reversible, long-acting depot; minimal injection frequency	Structural instability; untested immunogenicity	Pre-clinical (rodent)

## Data Availability

No new data were created or analyzed in this study.
